# Safety Profile and Enzymatic Specificity of *Aspergillus cristatus* CCNH008 Characterized by Genomic Analysis

**DOI:** 10.3390/jof12100759

**Published:** 2026-10-09

**Authors:** Yongming Duan, Xinyi Chen, Jiahao Huang, Juan Yang, Zichen He, Zhengyuan Zhai, Yanling Hao, Xiaowei Zheng

**Affiliations:** 1 Nutrition & Health Research Institute, COFCO Corporation, Beijing 102209, China; 2 Key Laboratory of Precision Nutrition and Food Quality, Department of Nutrition and Health, China Agricultural University, Beijing 100193, China; 3 College of Food Science and Nutritional Engineering, China Agricultural University, Beijing 100083, China; zhaizy@cau.edu.cn

**Keywords:** *Aspergillus cristatus*, comparative genomics, carbohydrate-related enzymes, genotype–phenotype discordance, antifungal susceptibility

## Abstract

The filamentous fungus *Aspergillus cristatus* is the principal fermentative microorganism that defines the quality of Fuzhuan brick tea (FBT). However, its specific enzymatic repertoire has not been systematically characterized at the genome level, limiting the broader biotechnological deployment of this fungus. Here we combined whole-genome sequencing, comparative genomic profiling against four well-characterized *Aspergillus* species (*Aspergillus niger*, *Aspergillus oryzae*, *Aspergillus nidulans*, and *Aspergillus fumigatus*), CAZyme annotation, and an empirical 19-enzyme activity panel benchmarked against a 50-strain *Aspergillus* reference set to define the enzymatic specificity of *A. cristatus* strain CCNH008. The 28.1 Mb hybrid assembly was the most compact genome among the five compared species and encoded the smallest CAZyme repertoire of the compared genomes, with a strongly restrained pectinolytic complement. Enzymatic profiling revealed a distinctive phenotype characterized by moderate cellulolytic capacity, marked polyphenol oxidase and protease activities, and a genotype–phenotype discordance in which pectinase activity ranks among the highest in the 50-strain panel despite a limited predicted pectinolytic gene complement. Comparative genomic profiling positioned CCNH008 between the lignocellulose-degrading specialist *A. niger* and the starch-adapted food fungus *A. oryzae*, consistent with adaptation to the unique chemical environment of tea leaves. The safety profile was assessed by in silico PHI/DFVF virulence factor screening and in vitro antifungal susceptibility testing. These findings establish a genomic and enzymatic foundation for the rational exploitation of *A. cristatus* in tea fermentation and related food biotechnology applications.

## 1. Introduction

Chinese dark teas, including Fuzhuan brick tea (FBT), represent a class of post-fermented teas whose distinctive quality arises from microbial-driven biotransformation of tea leaf substrates [1,2]. Tea leaves present a complex chemical matrix whose major macromolecular components include polyphenols (20–30% of dry weight), proteins (20–25%), cellulose (10–15%), hemicellulose (5–10%), pectin (2–5%), and starch (1–5%) [3,4]. FBT production involves a distinctive solid-state fermentation in which a succession of microorganisms colonizes the compressed tea leaves [1,2,3,4]. Among the diverse fungal community, *Aspergillus cristatus* is recognized as the key fermentative microorganism, and its abundance serves as a primary indicator of product quality [4]. Mechanistically, FBT fermentation proceeds through the secretion of extracellular enzymes that degrade the macromolecular constituents of tea leaves including cell wall polysaccharides, proteins, and polyphenols, thereby transforming the chemical and organoleptic properties of the tea substrate.

The ability to enzymatically deconstruct plant cell wall polysaccharides determines the ecological niche of a filamentous fungus in solid-substrate fermentation. Fungi deploy a diverse repertoire of carbohydrate-active enzymes (CAZymes), encompassing glycoside hydrolases (GHs), polysaccharide lyases (PLs), carbohydrate esterases (CEs), auxiliary activities (AAs), glycosyltransferases (GTs), and carbohydrate-binding modules (CBMs), to depolymerize structurally diverse plant polysaccharides [5]. The composition and abundance of CAZyme-encoding genes in a fungal genome largely dictate which substrates the organism can efficiently utilize. Comparative genomic studies across the genus *Aspergillus* have revealed considerable diversity in CAZyme content, reflecting species-specific adaptations to distinct ecological niches [6]. For instance, *A. niger* possesses an expanded set of pectinolytic and cellulolytic enzymes suited to its saprophytic lifestyle on fruits and vegetables [7], whereas *A. oryzae* shows enrichment in starch-degrading enzymes consistent with its domestication on starch-rich substrates [8]. Critically, closely related fungi can employ markedly different enzymatic strategies to degrade the same plant biomass, indicating that taxonomic proximity does not predict functional equivalence [9].

The genomic and enzymatic features of *A. cristatus* remain critical gaps in our understanding of its strain-level specialization. A draft genome of the FBT-fermenting strain *A. cristatus* GZ01, generated from Illumina short reads alone (27.9 Mb; 168 contigs/68 scaffolds; scaffold N50 ≈ 2.3 Mb; 10,136 predicted genes), was reported by Ge et al. [10], providing the first overview of the genetic repertoire of this species. To obtain a more contiguous and complete strain-level resource, we sequenced *A. cristatus* CCNH008 using a hybrid Illumina NovaSeq/PacBio Sequel strategy, yielding a 28.1 Mb assembly with an N50 of 3,578,266 bp. More recently, Wang et al. [11] demonstrated that *A. cristatus*-derived extracellular enzymes are the principal contributors to the characteristic aroma profile of FBT, establishing a direct functional link between fungal enzyme secretion and tea quality. However, no study to date has integrated genome-wide CAZyme predictions with systematic enzyme activity measurements benchmarked against a multi-strain *Aspergillus* reference panel to empirically define the enzyme specialization of *A. cristatus*.

In this study, we characterized *A. cristatus* strain CCNH008 isolated from FBT by combining whole-genome sequencing with comparative genomic profiling against four representative *Aspergillus* species (*A. niger*, *A. oryzae*, *A. nidulans*, and *A. fumigatus*). After annotating the CAZyme repertoire of CCNH008, we measured 19 extracellular enzyme activities and benchmarked them against a 50-strain *Aspergillus* reference panel, enabling direct comparison of genomic predictions with actual enzymatic phenotypes. A parallel safety assessment integrated in silico virulence factor screening with in vitro antifungal susceptibility testing.

## 2. Materials and Methods

### 2.1. Isolation and Molecular Characterization of the Fungal Strain

Fungal spores were enriched from FBT products and dilution-plated onto potato dextrose agar (PDA) medium supplemented with 100 µg/mL penicillin and 100 µg/mL streptomycin. The plates were incubated at 25 °C for 7 days. Distinct colonies were selected based on phenotypic traits including texture, color, zonation, sporulation, and colony diameter and subjected to purification. For microscopic analysis, a small portion of the fungal growth was mounted with lactophenol cotton blue on a clean slide and examined under a light microscope using a ×40 objective lens; isolates were characterized and identified using standard taxonomic guides.

For molecular identification, genomic DNA was extracted from fungal mycelia using the DNeasy Plant Mini Kit (Qiagen, Hilden, Germany). Two barcoding loci were amplified: the internal transcribed spacer (ITS) region with primers 5′-CTTGGTCATTTAGAGGAAGTAA-3′ and 5′-GCTGCGTTCTTCATCGATGC-3′, and the β-tubulin gene (BenA) with primers 5′-GGTAACCAAATCGGTGCTGCTTTC-3′ and 5′-ACCCTCAGTGTAGTGACCCTTGGC-3′ [12,13]. The 20 µL PCR mixture contained 1 µL genomic DNA template, 0.2 µL DNA polymerase, 0.5 µL each of forward/reverse primers, 1 µL dNTPs (2.5 mM each), and sterile double-distilled water to a final volume. The thermocycling protocol comprised initial denaturation at 94 °C for 5 min; 35 cycles of denaturation (94 °C, 45 s), annealing (55 °C, 45 s), and extension (72 °C, 1 min); and a final extension at 72 °C for 10 min. Sequences were aligned with the Clustal W algorithm in MEGA 5 and compared to reference sequences in the NCBI GenBank database via BLASTn V2.17.0. All consensus sequences were deposited in GenBank with unique accession numbers. Evolutionary relationships were inferred using the Maximum Likelihood method based on the Tamura–Nei model (MEGA 5) with 1000 bootstrap replicates.

### 2.2. Genome Sequencing and Gene Functional Analysis

Genomic DNA was isolated from fungal mycelia obtained from Potato Dextrose Broth (PDB) cultures using the DNeasy Plant Mini Kit (QIAGEN, Germany). DNA purity and integrity were assessed by agarose gel electrophoresis and quantified using Qubit fluorometry. Two sequencing libraries were constructed: a 350 bp Illumina library prepared with the NEBNext^®^ Ultra™ DNA Library Prep Kit (NEB, Ipswich, MA, USA) and a 20 kb SMRTbell™ library constructed using the SMRTbell™ Template Prep Kit (Pacific Biosciences, Menlo Park, CA, USA). Sequencing was performed at WEKEMO (Shenzhen, China), generating 4,090,413 paired-end (PE150) short reads (Illumina NovaSeq, San Diego, CA, USA) and 1,336,308 long reads (PacBio Sequel). Raw reads were quality-filtered using Trimmomatic v0.40 (Illumina) and default PacBio quality thresholds. De novo assembly was performed using Canu v2.2 (default parameters). The assembly was iteratively polished with Pilon (Illumina data) and Gcpp (PacBio data), and haplotypic duplications were removed with Purge_dups to produce the final assembly.

Protein-coding genes were predicted using Augustus v3.3 with species-specific parameters trained on *Aspergillus*. Repetitive elements were annotated using RepeatMasker v4.0.7 (Dfam 3.1 library) and Tandem Repeats Finder (TRF) v4.09. tRNAs were predicted with tRNAscan-SE v2.0, and small non-coding RNAs were identified by screening against the Rfam database v14.0 using cmsearch (E-value ≤ 0.01). Functional annotation was performed by assigning Gene Ontology (GO) terms, mapping KEGG pathways, and using KOG (Eukaryotic Orthologous Groups) for functional categorization. Protein domains were analyzed using the Pfam database v32.0. Carbohydrate-active enzymes were annotated via the CAZy database [5]. Secondary metabolite biosynthetic gene clusters (BGCs) were predicted using antiSMASH v7.0 with default fungal parameters. Pathogenicity-related genes were systematically annotated through the Pathogen-Host Interactions (PHI) database and the Database of Fungal Virulence Factors (DFVF). Assembly completeness was indirectly supported by the N50 (3,578,266 bp) and the gene-coverage metric (predicted coding sequences covering 57.7% of the assembly); a formal Benchmarking Universal Single-Copy Orthologs (BUSCO) assessment against fungi_odb10 was not performed at the time of assembly and will be included in a future genome update.

### 2.3. Pathogenicity Evaluation and Antifungal Susceptibility Testing

The minimum inhibitory concentrations (MICs) of seven antifungal agents against *A. cristatus* CCNH008 were determined using the broth microdilution method according to Clinical and Laboratory Standards Institute (CLSI) guidelines (M38-Ed4) [14]. Quality control was performed using the reference strain *Aspergillus fumigatus* ATCC 204305. The MIC values obtained for the QC strain fell within the established acceptable ranges (amphotericin B, 0.5–4 µg/mL; itraconazole, 0.06–0.5 µg/mL; voriconazole, 0.25–1 µg/mL; posaconazole, 0.125–0.5 µg/mL) at 48 h, confirming the validity of the susceptibility testing system [15]. Briefly, conidial suspensions were prepared in Roswell Park Memorial Institute (RPMI) 1640 medium (pH 7.0) and adjusted to 0.4–5 × 10^4^ CFU/mL, then dispensed into 96-well microplates containing serial two-fold dilutions of itraconazole, voriconazole, caspofungin, amphotericin B, fluconazole, ketoconazole, and flucytosine. After incubation at 35 °C for 48–72 h, MIC endpoints were defined as the lowest drug concentration showing complete visual inhibition. In silico virulence factor prediction was performed by systematic BLAST-based annotation against the PHI and DFVF databases, applying stringent filtering thresholds (E-value < 1 × 10^−5^, bit score > 200, identity > 85%, alignment length > 200 bp, gene coverage > 60%); these thresholds were adapted from the conservative filtering strategy employed in comparable fungal genome safety assessments [10], with minor modifications, and were selected to favor specificity at the expense of sensitivity, thereby retaining only high-confidence virulence homologs.

### 2.4. Comparative Genomic Profiling

For comparative analysis, the genome of *A. cristatus* CCNH008 was compared with four publicly available *Aspergillus* reference genomes: *A. niger* CBS 513.88 (GCA_000002855.1) [16], *A. oryzae* RIB40 (GCA_000184455.2) [8], *A. nidulans* FGSC A4 (GCA_000149205.2) [17], and *A. fumigatus* Af293 (GCA_000002655.1) [18]. General genomic features, including genome size, GC content, and gene counts, were obtained from the respective genome publications [8,16,17,18]. To ensure like-for-like family-level comparisons, CAZyme gene counts for all five species were computed under a single uniform annotation pipeline (hmmscan against the dbCAN HMM database V9, with gene-level counts obtained by collapsing domain assignments to unique gene loci); family nomenclature follows the CAZy database [5]. These uniform-pipeline counts supersede the service-provider CAZyme annotation originally delivered for CCNH008 and are tabulated in Supplementary Table S6. The proteome of the previously published *A. cristatus* strain GZ01 (NCBI protein annotation of assembly GCA_001717485.1) was annotated with the same uniform pipeline to enable species-level comparison (Section 3.3). For CCNH008, CAZyme domain assignments were derived from hmmscan searches against the dbCAN HMM database (version 9), and gene-level counts were obtained by collapsing the assignments to unique gene loci; the curated inventory of key CAZyme genes is provided in Supplementary Table S5. Secondary metabolite BGCs were predicted using antiSMASH v7.0 with default fungal parameters. Comparative analysis of CAZyme family distributions focused on plant cell wall–degrading enzyme families (GH5, GH6, GH7, GH10, GH11, GH12, GH28, GH43, PL1, PL3, PL4, AA1, AA2, AA9, CBM1, and CBM67), following the framework of de Vries et al. [6]. Core carbon metabolism pathway completeness was assessed by ortholog presence across the five genomes. Ortholog clustering across the five proteomes was performed from an all-vs-all sequence search (DIAMOND v2.2.7, E ≤ 0.001) followed by Markov-cluster grouping, yielding 22,784 orthogroups (8516 containing more than one gene, 8139 with genes from more than one species); family-level gene counts for every species were computed under a single uniform CAZy-family annotation pipeline (hmmscan against the dbCAN HMM database V9), and pairwise dN/dS (Nei–Gojobori) was estimated for orthologous pectinase-related genes (see Section 3.3 and Supplementary Table S6).

### 2.5. Fungal Culture and Enzyme Activity Assays

The filamentous fungal strain was initially activated on PDA medium for two generations, then transferred to malt sporulation medium for 5–7 days. Spores were harvested by washing plates with sterile saline, filtering out mycelia, and adjusting to 10^8^ CFU/mL using a turbidimeter. Aliquots of 1 mL of this suspension were used to inoculate 100 mL of PDB medium in 300 mL Erlenmeyer flasks, and cultured for 4 days at 25 °C with shaking at 180 rpm, yielding a final concentration of 1 × 10^8^ CFU/mL. Three biological replicates were prepared on independent days, and each biological replicate was assayed in three technical replicates (*n* = 3 + 3). The supernatant was collected as a crude enzyme extract and stored at 4 °C for ≤1 week, while mycelial pellets were dried to constant weight at 60 °C for dry-weight determination. One enzyme unit (U) was uniformly defined as the amount of enzyme producing 1 µmol of product per minute under the assay-specific optimal conditions. Enzyme activities were calculated as units per gram of fungal dry weight (U/g DW); the substrate-specific operational definitions in Supplementary Table S3 are expressed in this unit system.

A 50-strain *Aspergillus* reference panel was established in-house to enable cross-species percentile benchmarking. The complete species composition and strain identifiers are provided in Supplementary Table S2. The 19-enzyme panel covered lignocellulose-degrading enzymes (cellulase, β-glucosidase, neutral xylanase, acid xylanase), polyphenol/lignin-modifying enzymes (polyphenol oxidase, tannase), starch/glycogen-degrading enzymes (α-amylase, β-amylase, glucoamylase), pectin-degrading enzymes (pectinase), proteases/peptidases (neutral protease, alkaline protease, aminopeptidase), and additional nutritional enzymes (Supplementary Tables S2 and S3). All strains in the reference panel were cultured and assayed under identical conditions in the same laboratory; inter-batch variability was controlled by including a reference strain (*A. niger* ATCC 1015) on every microplate. Raw enzyme activities (U/g DW) for each test strain were divided by the same-batch activity of the reference strain and multiplied by the panel-wide median reference-strain activity to yield normalized, batch-corrected values; this multiplicative scaling preserves the rank ordering of strains while removing systematic batch offsets. Percentile assignment followed the standard empirical percentile-rank rule: for each enzyme, the percentile of a strain equals 100 × (number of panel strains whose normalized activity is less than or equal to that of the strain)/50; the panel maximum therefore corresponds to the 100th percentile (e.g., pectinase, 50/50), one strain ranking above gives the 98th percentile (49/50, e.g., α-/β-amylase and polyphenol oxidase), and a mid-panel value gives intermediate percentiles (e.g., cellulase, 29/50 = 58th). When an enzyme was not detected in CCNH008 (activity below the detection limit, recorded as zero), the tie-inclusive formula yields the percentile of the zero-value tie and the enzyme is flagged as astericks in Section 3.4, which was not detected. Assay chemistries followed established colorimetric frameworks: the dinitrosalicylic acid (DNS) reducing-sugar assay [19] for amylases, xylanases, glucoamylase, and pectinase; the casein-based, tyrosine-equivalent protease assay [20,21]; the copper-soap colorimetric assay [22] for lipase; and carboxymethyl-cellulose (anthrone colorimetry [23]) and p-nitrophenyl-substrate assays [24] for cellulase and glucosidases; the feruloyl esterase assay followed the p-nitrophenyl-ferulate method [25]. Full substrate-specific definitions, calibration curves, and protocols for all 19 enzymes are provided in Supplementary Table S3, with the normalized activity values of the 50-strain panel in Supplementary Table S2.

## 3. Results

### 3.1. Morphological and Molecular Identification of Fungal Aspergillus cristatus CCNH008

**Figure 1:**
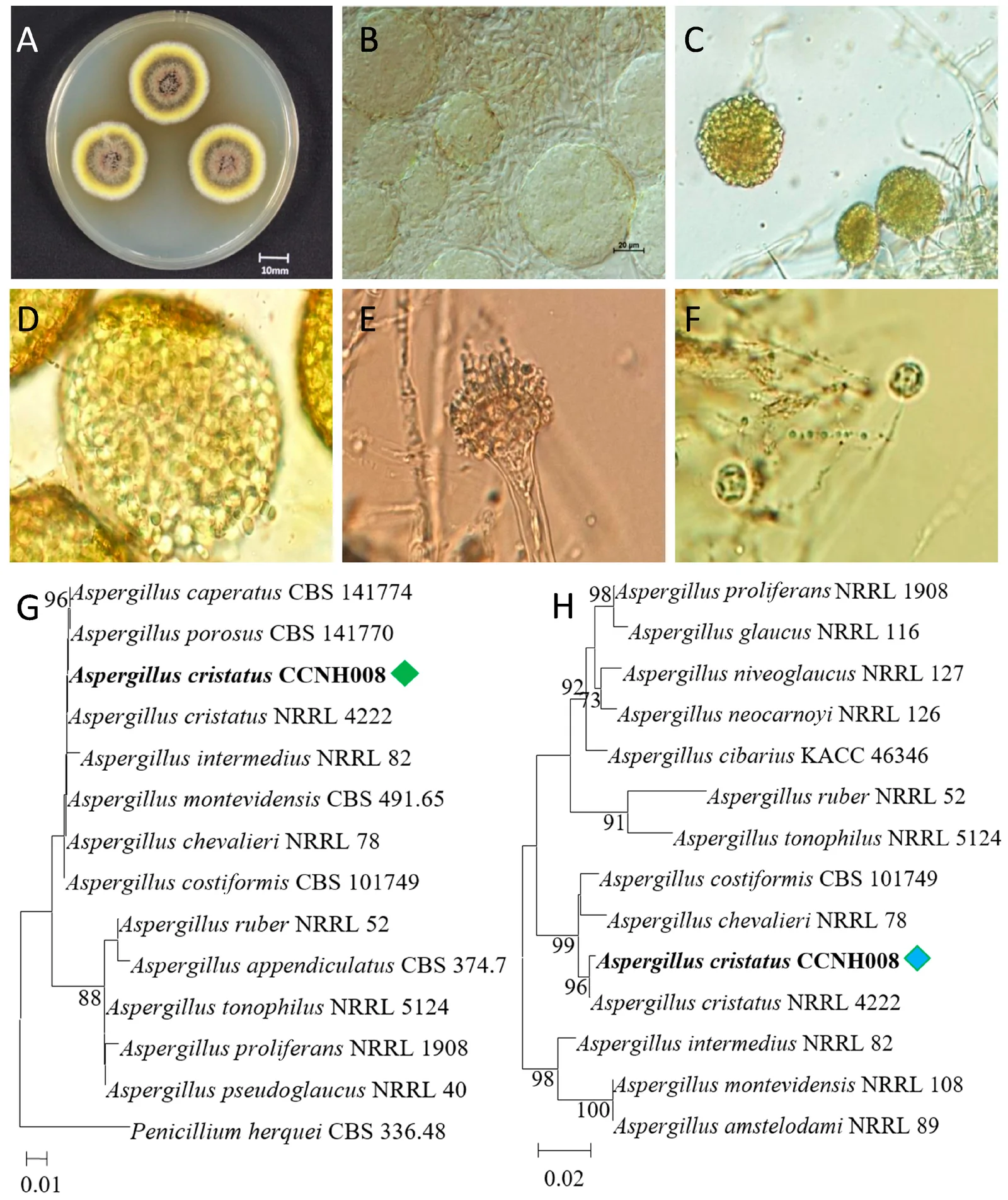
Morphological characterization and DNA-based phylogenetic identification of *Aspergillus cristatus* CCNH008. After 7 days of incubation at 25 °C on PDA, the fungal colony displayed a brown center surrounded by a pale yellow periphery (**A**). Morphological features show a raised, velvety colony center with fine, interwoven yellow mycelia and abundant yellow cleistothecia (20–110 µm in diameter) embedded within the hyphal network (**B**–**F**). Subglobose to ellipsoidal asci (6–12 µm in length) contain ascospores. Phylogenetic trees based on the ITS region ((**G**), CCNH008 marked by green square) and BenA gene ((**H**), CCNH008 marked by blue square) robustly placed the isolate within *A. cristatus* (1000 bootstrap replicates).

After seven days of incubation at 25 °C on PDA, the fungal colony displayed a brown center surrounded by a pale yellow periphery (Figure 1A). Numerous yellow cleistothecia (20–110 µm in diameter) were enmeshed within a network of hyaline, smooth-surfaced hyphae, with subglobose to ellipsoidal cleistothecia and subglobose asci (Figure 1B–D). Conidial structures were also observed, characterized by smooth conidiophores bearing subglobose apical vesicles with uniseriate phialides and subglobose conidia (Figure 1E,F). These morphological characteristics indicated that the fungus possesses both sexual and asexual reproductive strategies.

For species-level identification, sequence analysis of the internal transcribed spacer of ribosomal DNA (ITS rDNA) and the β-tubulin gene (BenA) was conducted [12,13]. The ITS rDNA sequence (510 bp) showed 100% identity with five *Aspergillus* species. Phylogenetic analysis using the neighbor-joining method (1000 bootstrap replicates) placed the isolate closest to *A. cristatus* NRRL 4222, *A. caperatus* CBS 141774, and *A. porosus* CBS 141770 (Figure 1G). A BenA-based phylogenetic tree confirmed closest affinity to *A. cristatus* NRRL 4222 (Figure 1H). Based on the concordance between morphological characteristics and dual-locus sequence analysis, the fungal isolate was identified as *A. cristatus* and designated strain CCNH008.

### 3.2. Qualified Safety Profile of CCNH008 Evaluated by Virulence Factor Analysis and Antifungal Susceptibility Testing

**Figure 2:**
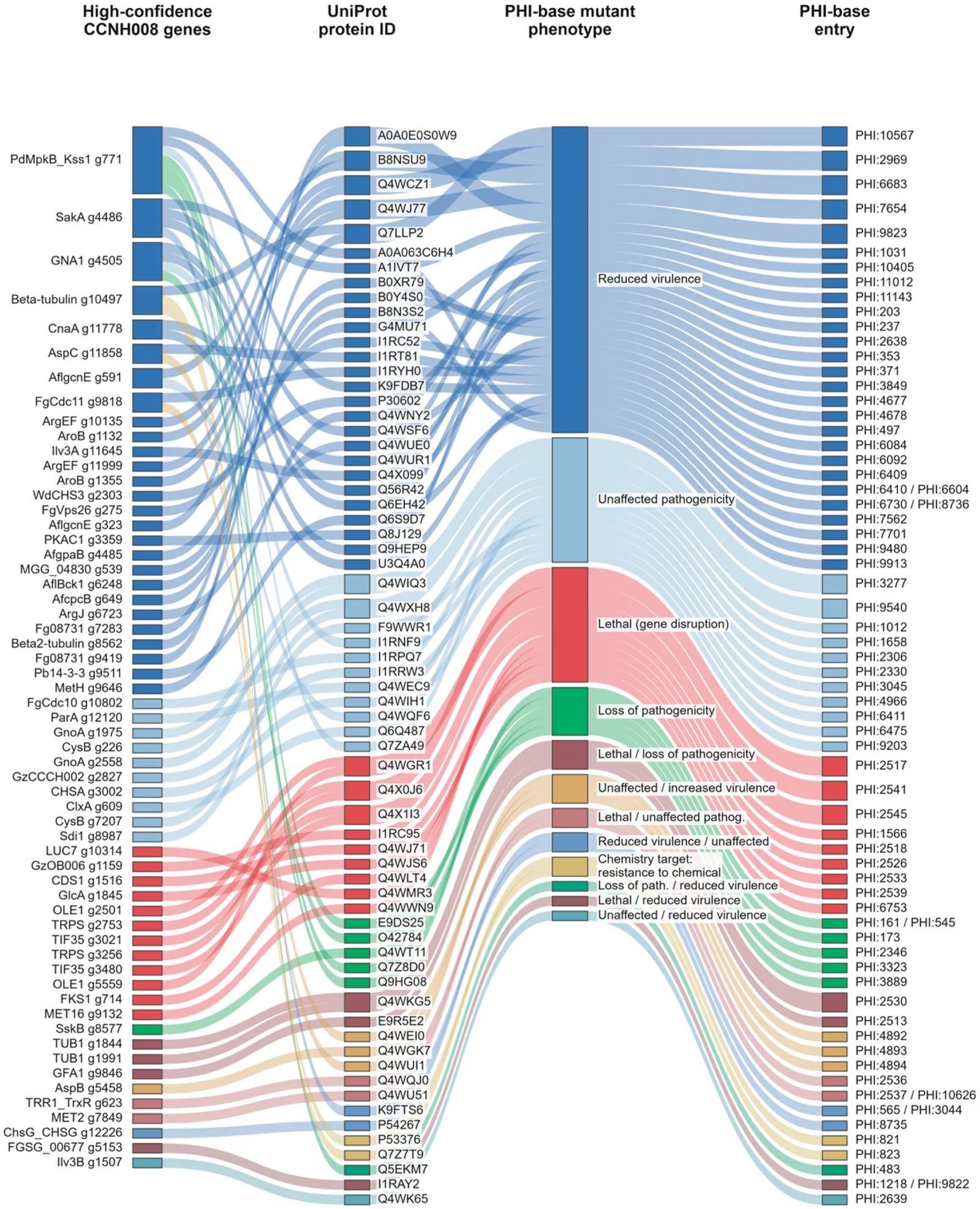
Integrated safety assessment of *A. cristatus* CCNH008 combining genomic and toxicological analyses. In silico virulence factor screening annotated 775 and 728 genes using the PHI and DFVF databases, respectively; after stringent filtering, 77 PHI and 56 DFVF high-confidence genes were retained, merged by gene/protein ID, and clustered by mutant phenotype. The alluvial layout is organized into four labelled sections: high-confidence CCNH008 genes (left), their UniProt protein IDs, PHI-base mutant-phenotype classes, and PHI-base entries (right). The vertical extent of each gene block reflects the number of retained hits assigned to that gene, and the curved ribbons follow the standard convention of the alluvial layout: each gene node sits at a different vertical position from its assigned phenotype class and PHI entry, so assignments are drawn as smooth curves whose slope and crossings visualize how many genes converge on each phenotype class rather than encoding any additional variable.

To evaluate the pathogenic potential of *A. cristatus* CCNH008, in silico virulence factor prediction was performed by systematic BLAST-based annotation against the PHI and DFVF databases. After applying stringent filtering thresholds (E-value < 1 × 10^−50^, bit score > 200, identity > 85%, alignment length > 200 bp, gene coverage > 60%), 77 and 56 high-confidence virulence homologs were retained from the PHI and DFVF databases, respectively (Figure 2). The merged set clustered into lethal-disruption genes (e.g., LUC7, FKS1), virulence-related genes (e.g., PKAC1, BMP1), and dual-function essential/virulence genes (e.g., TUB1, GFA1); Tub2 was associated solely with chemical resistance. Notably, antiSMASH analysis identified diverse secondary-metabolite biosynthetic gene clusters, including Type I Polyketide Synthase (T1PKS), Non-Ribosomal Peptide Synthetase (NRPS), terpene, siderophore, indole, and NAPAA, but no known mycotoxin biosynthesis clusters (aflatoxin, ochratoxin, fumonisin) were detected, supporting the safety profile of CCNH008 in food fermentation applications, consistent with chemical studies that isolated bioactive anthraquinones from FBT-derived *A. cristatus* [26]. Detailed GO, KEGG, and KOG functional annotations are provided in Supplementary Table S4.

Antifungal susceptibility testing against seven agents was performed using the broth microdilution method according to CLSI M38-Ed4 guidelines (Table 1). The concurrent QC results for *A. fumigatus* ATCC 204305 fell within the established quality-control ranges, indicating that the broth microdilution procedure was performed under acceptable conditions [15]. CCNH008 was classified as susceptible to itraconazole, voriconazole, caspofungin, and amphotericin B. Formal categorization for fluconazole, ketoconazole, and flucytosine was precluded by the absence of CLSI breakpoints for *Aspergillus* spp. However, the elevated fluconazole MIC is consistent with the intrinsic azole resistance widely reported in the genus [27,28,29]. The combination of genomic absence of acquired resistance determinants and phenotypic susceptibility to major antifungal classes provides convergent evidence supporting a qualified low-risk safety profile.

### 3.3. A Compact and Contracted CAZyme Repertoire in CCNH008 Revealed by Comparative Genomic Analysis

To profile the carbohydrate-active enzyme repertoire of *A. cristatus* CCNH008, we annotated CAZyme genes under a single uniform pipeline (dbCAN HMM database V9) for all five species and compared family-level gene counts against four reference *Aspergillus* species (*A. niger*, *A. oryzae*, *A. nidulans*, and *A. fumigatus*, Table 2).

**Figure 3:**
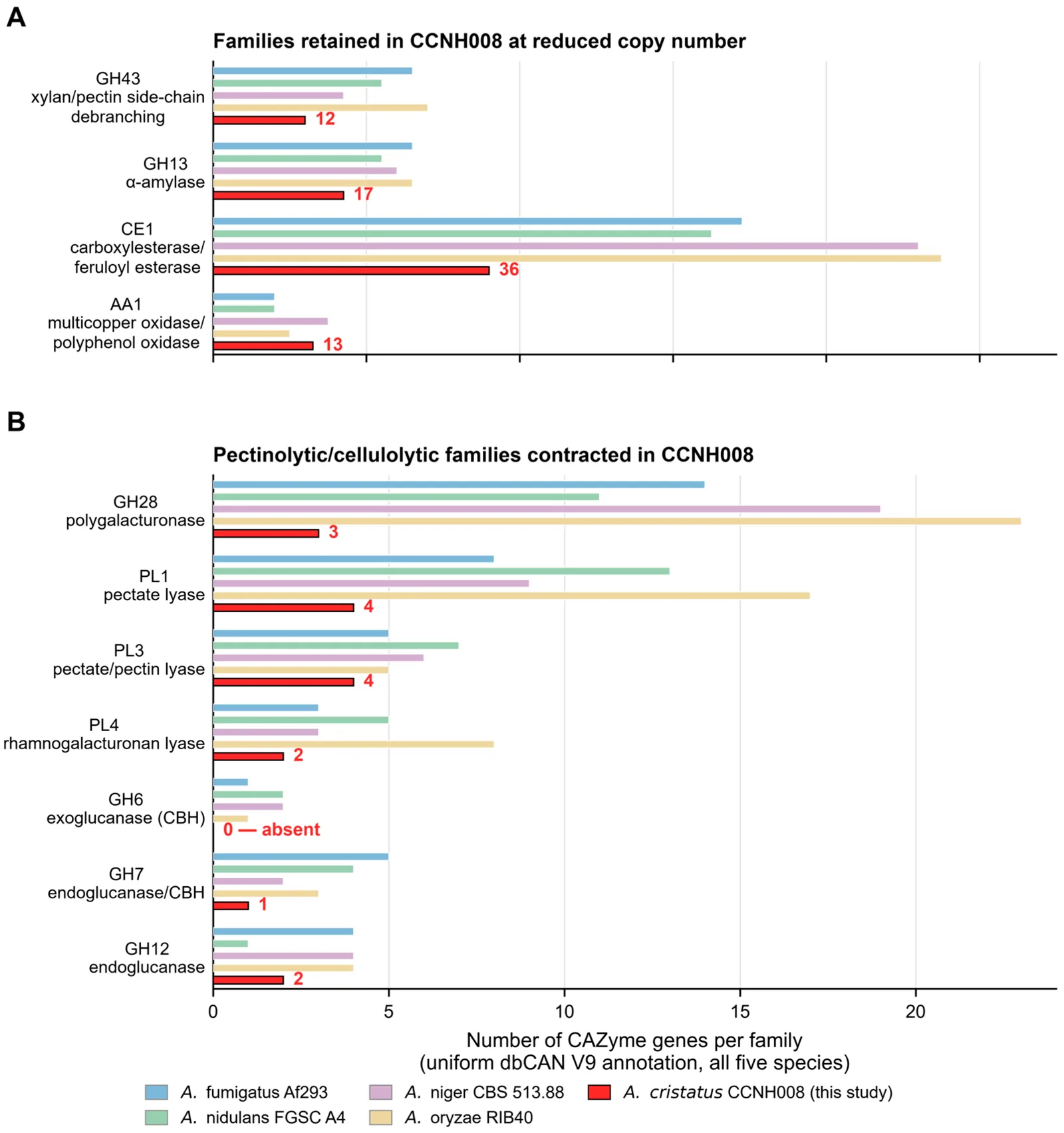
Comparative CAZyme family profiles of *Aspergillus cristatus* CCNH008 and four reference *Aspergillus* genomes, all annotated under a single uniform pipeline. Bars show family-level CAZyme gene counts for the five species; CCNH008 was highlighted in red with counts labeled at the bar ends. (**A**) Families retained in CCNH008 at reduced but detectable copy number: GH43 (xylan/pectin side-chain debranching enzymes), GH13 (α-amylases), CE1 (carboxylesterases/feruloyl esterases), and AA1 (multicopper oxidases/polyphenol oxidases). (**B**) Pectinolytic and cellulolytic families contracted in CCNH008: GH28 (polygalacturonases), PL1, PL3, and PL4 (pectate/pectin lyases), GH6 (exoglucanases), and GH7/GH12 (endoglucanases). Note the complete absence of GH6 in CCNH008 (0 genes), whereas one to two copies occur in each reference species. Panels (**A**,**B**) are drawn on different *x*-axis scales.

The CCNH008 genome (28.1 Mb) encodes 10,599 genes, representing the most compact genome among the five species (Figure 3). CAZyme annotation under the uniform dbCAN V9 pipeline identified 1703 domain assignments collapsing to 592 unique CAZyme-encoding genes, which was the smallest repertoire among the five species (Table 2 and Table S5). Glycoside Hydrolases (GHs) accounted for 35% (207 genes) and Auxiliary Activities (AAs) for 22% (132 genes), whereas Polysaccharide Lyases (PLs, 25 genes), Carbohydrate Esterases (CEs, 73 genes), and Carbohydrate-Binding Modules (CBMs, 33 genes) were less abundant.

Under the uniform gene-level annotation, no examined family showed a copy-number excess in CCNH008 (Figure 3 and Tables S5 and S6). The GH13 family (α-amylases) comprised 17 genes, approaching the complements of the reference species (22–26 genes), and the AA1 family (multicopper oxidases/polyphenol oxidases) was represented by 13 genes, exceeded only by *A. niger* (15 genes). The CE1 family (carboxylesterases/feruloyl esterases) contained 36 genes and the GH43 family (xylan/pectin side-chain debranching enzymes) 12 genes, both the smallest inventories among the five species.

Conversely, the core pectinolytic and cellulolytic families were strongly contracted (Figure 3). The GH6 family (exoglucanases) was entirely absent (0 genes), whereas one to two copies occur in each reference species. The GH28 family (polygalacturonases) comprised only 3 genes, versus 11–23 in the references, and the PL1, PL3, and PL4 families (pectate/pectin lyases) were represented by 4, 4, and 2 genes, respectively. The GH7 and GH12 families (endoglucanases) were represented by 1 and 2 genes, respectively, the lowest and second-lowest inventories in the comparison. Ortholog clustering across the five proteomes provided a genome-wide framework for testing whether the family-level differences reflect statistically supported lineage-specific expansions (Supplementary Table S6). Gene counts within orthogroups were tested for expansion or contraction in CCNH008 for 327 CAZyme families (Fisher’s exact test with Benjamini–Hochberg correction); no family remained significant after correction, and the pectinase-relevant families were uniformly contracted rather than expanded. None of the retained pectinolytic genes in CCNH008 was tandemly duplicated. Pairwise dN/dS (Nei–Gojobori) estimation for 166 orthologous pectinase-related gene pairs spanning 11 families yielded values of 0.024–0.197 (median 0.074), with no pair exceeding ω = 1, indicating pervasive purifying selection and no evidence of positive selection on the retained pectinolytic genes (Supplementary Table S6).

In summary, comparative genomics under a uniform annotation pipeline revealed a compact, consistently contracted CAZyme repertoire in CCNH008. Every examined plant-cell-wall-degrading family was present at the lowest or near-lowest copy number among the five species. Moreover, ortholog-level analyses detected no significant family expansion and no positive selection acting on the retained pectinolytic genes.

### 3.4. Pectinase Activity Ranks Highest in Enzymatic Phenotyping Panel

**Figure 4:**
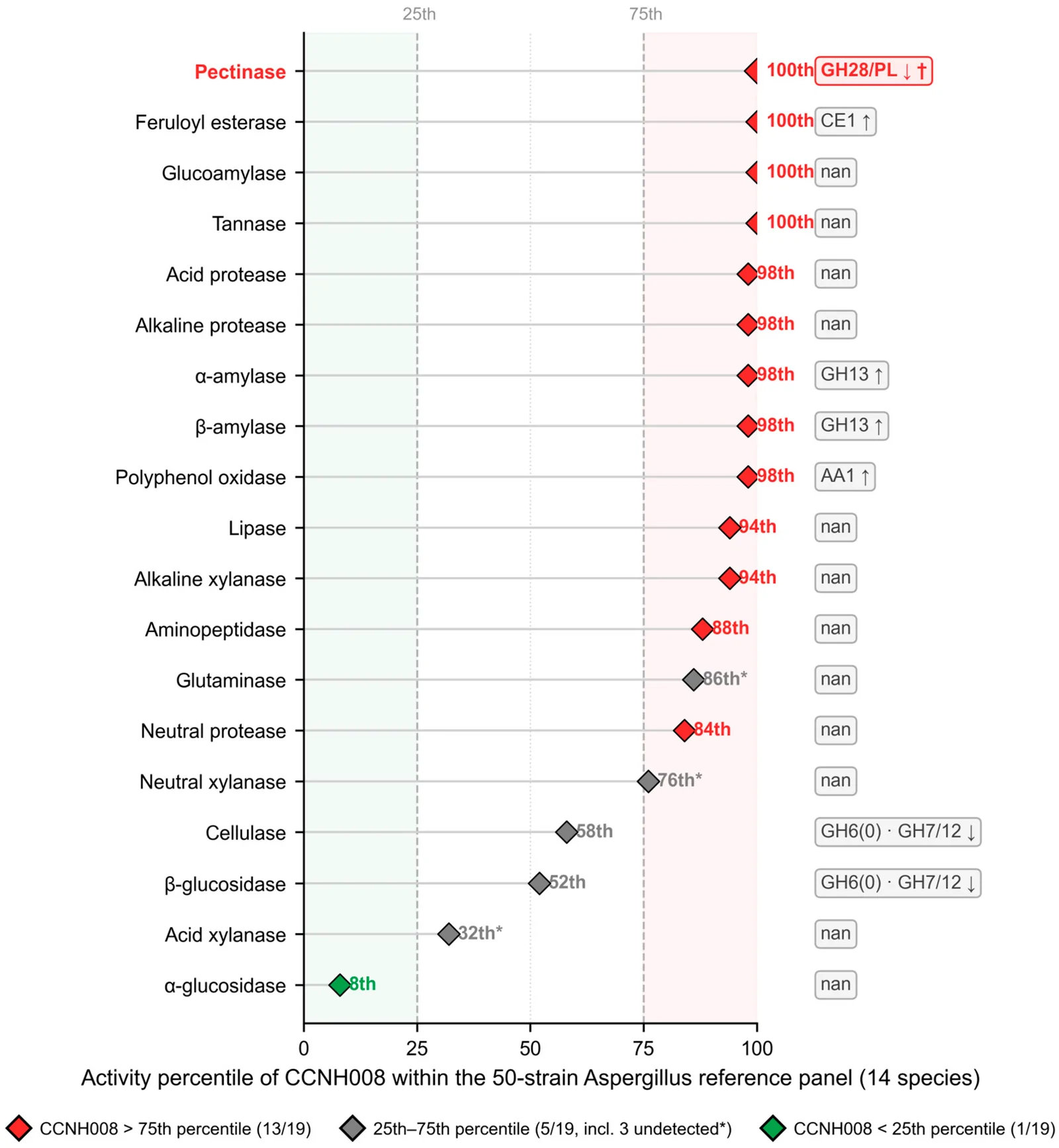
Extracellular enzyme activity profile of *A. cristatus* CCNH008 benchmarked against a 50-strain *Aspergillus* reference panel spanning 14 species. Each row shows the activity percentile of CCNH008 for one of the 19 tested extracellular enzymes; activities were normalized batch-to-batch using the internal reference strain *A. niger* ATCC 1015 before percentile ranking. Diamonds are colored by percentile band: red, above the 75th percentile (13/19 enzymes); gray, within the 25th–75th percentile or tied at zero because activity was not detected (5/19); green, below the 25th percentile (1/19). Dashed lines mark the 25th and 75th percentiles. Tags on the right link enzymes to the CAZyme families. An asterisk (*) after a percentile indicates that the activity of that enzyme was not detected in CCNH008.

To validate genomic predictions, we measured the extracellular activities of 19 enzymes for CCNH008 and benchmarked the results against a 50-strain *Aspergillus* reference panel spanning 14 species. Activity values were normalized batch-to-batch using an internal reference strain (*A. niger* ATCC 1015) and expressed as percentiles within the panel (Figure 4).

Pectinase activity ranked first (100th percentile) among the 50 strains, representing the highest rank across all 19 tested enzymes. The majority of enzyme activities were consistent with genomic predictions. The high-ranking amylases (α- and β-amylase, both 98th percentile) corresponded to the relatively retained GH13 complement (17 genes). The high polyphenol oxidase activity (98th percentile) was consistent with the comparatively retained AA1 family (13 genes). The rank-1 feruloyl esterase activity coincided with the presence of a CE1 complement (36 genes), although its copy number was the lowest among the five species. The moderate cellulase and β-glucosidase activities (58th and 52nd percentiles, respectively) were consistent with the absence of GH6 and the single-copy GH7/two-copy GH12 representation.

Despite the strongly contracted genomic inventory of pectinolytic families (GH28: 3 genes; PL1/PL3/PL4: 4, 4, and 2 genes; GH43: 12 genes), pectinase activity ranked first (100th percentile), showing a genotype–phenotype discordance, which was not observed for any other enzyme in the panel.

In summary, enzymatic phenotyping placed CCNH008 as a high performer for pectinase, amylases, esterases, oxidases, and proteases, with moderate cellulolytic activity. The pectinase result is the most notable deviation from the genomic prediction.

## 4. Discussion

The taxonomic identity of “golden-flower” fungi from Fuzhuan brick tea has been historically debated, with classifications spanning *Eurotium*, *Aspergillus*, and related genera [3,4]. In this study, we employed a dual-marker molecular identification strategy combining the internal transcribed spacer (ITS) region and the β-tubulin gene (BenA), both established barcoding loci for species-level discrimination within the genus *Aspergillus* [12,13]. The concordance between morphological characterization and microscopic features and phylogenetic analysis based on ITS and BenA sequences unambiguously placed strain CCNH008 within *A. cristatus*. This finding is consistent with the taxonomic assignments reported in recent genomic studies of tea-derived *Aspergillus* isolates [10] and reinforces the utility of the ITS + BenA dual-locus approach for resolving the identities of fermentation-associated *Aspergillus* strains. Whole-genome sequencing further corroborated this identification by providing genome-scale phylogenetic resolution, and the availability of a high-quality genome assembly enables phylogenomic approaches including genome-wide average nucleotide identity (ANI) and multi-locus sequence analysis incorporating additional protein-coding loci such as calmodulin (CaM) and RNA polymerase II second largest subunit (RPB2) to further refine the taxonomic framework for *A. cristatus* and its close relatives.

A notable genotype–phenotype discordance was observed for pectin degradation. Despite a limited repertoire of predicted pectinolytic CAZyme families (GH28, PL1, PL3, PL4), pectinase activity in CCNH008 ranked among the highest in the 50-strain reference panel. Under the uniform annotation pipeline, this repertoire is even more compact than initially annotated, sharpening the discordance. Comparative genomic profiling revealed that CCNH008 possesses the smallest genome within the compared species set, with a compact cellulase repertoire relative to *A. niger* and a restrained pectinolytic gene complement, features consistent with adaptation to tea leaves, in which pectin is a minor (2–5% of dry weight) but accessible wall component that is partly solubilized during the steaming and pressing steps of FBT processing, thereby releasing pectin-derived inducers during fermentation, rather than the fruit and vegetable substrates colonized by *A. niger* [7]. Its moderate CBM complement further suggests an enzymatic strategy adapted for partial degradation rather than complete biomass deconstruction. The conservation of core carbon metabolism pathways (glycolysis, TCA cycle, pentose phosphate pathway) across all five species confirms that interspecific functional divergence is concentrated in the auxiliary CAZyme arsenal rather than in central metabolism. Ortholog clustering across the five proteomes showed no CAZyme family to be significant after multiple-testing correction, and pairwise dN/dS estimation (166 pectinase ortholog pairs; median 0.074, none exceeding ω = 1) indicated that the few retained pectinolytic genes of CCNH008 are conserved and under purifying selection rather than products of recent lineage-specific expansion. In addition to canonical GH28/PL activities, the retained GH43 (12 genes) and CE1 (36 genes) complements could cooperate in pectin side-chain remodeling, and the high measured activity may reflect strong induction of these few genes under pectin-containing conditions rather than an expanded repertoire—hypotheses that require transcriptomic and biochemical validation. Synteny reconstruction and formal gain/loss modeling remain future work.

Within the compared set, phylogenetic divergence and CAZyme repertoire evolution are congruent. The plant-litter- and fruit-colonizing decomposer *A. niger* retains the largest genome (34.9 Mb) and the most complete plant-cell-wall degradation arsenal; the starch-adapted domesticated species *A. oryzae* shows GH13 expansion; and the pectinase producers of the genus (*A. niger*, *A. awamori*, *A. terreus*, *A. flavipes*, *A. flavus*) all harbor large GH28/PL complements [6,7]. *A. cristatus* CCNH008, with the smallest genome in the compared set, combines the smallest overall CAZyme repertoire with a uniform contraction of plant-cell-wall-degrading families, including core pectinolytic GH28 and PL1/PL3/PL4. Thus, transcriptomic profiling under pectin-inducing conditions, together with biochemical characterization of the few retained enzymes, would clarify how the observed pectinase activity is achieved; translating this enzyme profile into the actual chemical transformation of tea leaves during fermentation, and extending the comparison to additional *A. cristatus* isolates, such as GZ01 and JH-5 [30], to test whether CCNH008 is representative, are key directions for future work. Consistent with a species-level interpretation, the previously sequenced *A. cristatus* GZ01, annotated here under the same uniform pipeline (Table 2), displays the same contracted pectinolytic profile as CCNH008. Genome compaction is therefore a species-level feature rather than a hard size constraint, and the selective loss of plant-cell-wall-degrading families, combined with the tea-substrate lifestyle of *A. cristatus*, supports niche-driven repertoire trimming [6]. Gain/loss modeling over a denser *Aspergillus* sampling is planned as future work. More broadly, pairing genome-scale CAZyme prediction with an empirical multi-strain activity panel offers a transferable paradigm for genotype–phenotype-anchored enzyme discovery in non-model fermentation fungi.

In this study, we adopted a multi-layered approach combining genome-based virulence factor prediction with phenotypic antifungal susceptibility testing. The PHI and DFVF database analyses identified 77 and 56 high-confidence virulence-associated genes, respectively, including both lethal-disruption genes (LUC7, CDS1, OLE1, TRPS, FKS1) and virulence-related genes (PKAC1, BCG1, VMK1, BMP1) (Figure 2). This genomic screening approach offers a key advantage over conventional phenotypic pathogenicity testing alone. Moreover, the safety conclusion is further supported by a complementary in vivo toxicological evaluation of the same strain, which found no adverse effects in acute and 90-day oral toxicity studies or in genotoxicity assays [31]. Collectively, the genomic and phenotypic evidence supports a qualified low-risk safety profile for CCNH008 across the *A. cristatus* species.

## Figures and Tables

**Table 1:** Antifungal susceptibility of *A. cristatus* CCNH008. The minimum inhibitory concentration (MIC) of seven antifungal agents was determined using the broth microdilution method (CLSI M38-Ed4). Strain categorization as susceptible (S), intermediate (I), or resistant (R) was established following the interpretive criteria of the CLSI M38-Ed4 (2022) standard. NC, not categorized (no CLSI breakpoint available for *Aspergillus* spp.).

Antifungal Agents	MIC (μg/mL)	Susceptible (S)	Intermediate (I)	Resistant (R)	Determination
Itraconazole	0.5	≤0.5	1	≥2	S
Voriconazole	0.5	≤0.5	1	≥2	S
Caspofungin	0.0625	≤2	4	≥8	S
Amphotericin B	0.125	≤1	2	≥4	S
Fluconazole	16	NC	NC	NC	NC
Ketoconazole	0.25	NC	NC	NC	NC
5-Flucytosine	2	NC	NC	NC	NC

**Table 2:** Comparative genomic features of *A. cristatus* CCNH008, four representative *Aspergillus* species, and the previously sequenced *A. cristatus* GZ01. ^a^ CAZyme gene counts for all listed genomes computed under a single uniform annotation pipeline. ^b^ Predicted biosynthetic gene clusters of CCNH008 from antiSMASH analysis. BGC counts for the reference species and GZ01 are from their respective genome publications.

Species	Genome Size (Mb)	GC (%)	Gene Count	CAZyme Genes	SM BGCs
*A. cristatus* CCNH008 (This study)	28.1	49.75	10,599	592 ^a^	~41 ^b^
*A. niger* CBS 513.88	34.9	49.8	~14,165	936	~81
*A. oryzae* RIB40	37.2	48.4	~12,074	1087	~62
*A. nidulans* FGSC A4	30.1	49.9	~10,704	910	~71
*A. fumigatus* Af293	29.4	49.3	~9926	831	~36
*A. cristatus* GZ01	27.9	49.7	10,136	657 ^a^	~39

## Data Availability

All data generated or analyzed during this study are included in this published article and its Supplementary Files. The whole-genome sequencing data for *Aspergillus cristatus* CCNH008 have been deposited at the China National Center for Bioinformation (CNCB; https://www.cncb.ac.cn) under Project Accession PRJCA050413 and Sample Accession SAMC6087844. The live strain has been deposited in the China General Microbiological Culture Collection Center (CGMCC) under accession number CGMCC 41727. Reference genome assemblies used in the comparative analysis are publicly available from NCBI GenBank: *A. niger* CBS 513.88 (GCA_000002855.1), *A. oryzae* RIB40 (GCA_000184455.2), *A. nidulans* FGSC A4 (GCA_000149205.2), and *A. fumigatus* Af293 (GCA_000002655.1).

## Supplementary Materials

The following supporting information can be downloaded at: https://www.mdpi.com/article/10.3390/jof12100759/s1, Table S1: Gene information conclusion; Table S2: *Aspergillus* enzymes; Table S3: Enzyme activity definitions and assay protocols; Table S4: Gene functional annotations; Table S5: Key cazyme genes; Table S6: Ortholog analysis.
