# Supplementary material for: Safety Profile and Enzymatic Specificity of Aspergillus cristatus CCNH008 Characterized by Genomic Analysis
Source: J Fungi (Basel). 2026 Oct 9;12(10):759. doi: 10.3390/jof12100759 (PMC13706197; doi:10.3390/jof12100759)
Supplement: Supplementary file 1 [file jof-12-00759-s001.zip › Table S3.Enzyme_activity_definitions_and_assay_protocols.pdf]

Supplementary Table S3. Enzyme activity definitions, assay protocols, calibration, and data-presentation methods

1. General workflow and sample basis

The filamentous fungus was activated on potato dextrose agar (PDA) for two generations and transferred to malt sporulation medium for 5–7 days. Spores were harvested with sterile saline, filtered to remove mycelia, and adjusted to 10^8 CFU/mL. Aliquots (1 mL) were inoculated into 100 mL of potato dextrose broth (PDB) in 300 mL Erlenmeyer flasks and cultured for 4 days at 25 °C with shaking at 180 rpm (Methods 2.5). Three biological replicates were prepared on independent days and assayed in triplicate (n = 3 + 3). Cultures were centrifuged; the supernatant was collected as the crude enzyme extract, and the mycelial pellet was dried to constant weight at 60 °C to obtain the fungal dry weight (DW). All activities are expressed relative to fungal DW (per gram of dried mycelium) and stated explicitly in Methods 2.5.

2. Definition of the enzyme unit and specific activity

Because each of the 19 assays is calibrated against a different product-equivalent standard (Section 5, Table S3-2), the operational unit of each assay is documented per assay in Table S3-1. These operational units are related to the uniform U convention by the molar mass of the calibration standard and the assay time base. Critically, all cross-strain and cross-enzyme comparisons in the manuscript are based exclusively on the batch-corrected ratios and percentile ranks described in Section 3, which are ratios of identically calibrated measurements and are therefore invariant to the absolute unit convention.

3. Data analysis and presentation: batch correction and percentile assignment

All 50 panel strains, including CCNH008, were cultured and assayed under identical conditions in the same laboratory, and a reference strain (*A. niger* ATCC 1015) was included on every microplate to control inter-batch variability. Raw activity (per g DW) of each test strain was divided by the same-batch activity of the internal reference strain and multiplied by the panel-wide median reference-strain activity to yield normalized, batch-corrected values; this multiplicative scaling preserves the rank ordering of strains while removing systematic batch offsets.

Percentile assignment follows the standard empirical percentile-rank rule: for each enzyme, the percentile of a strain equals 100 × (number of panel strains whose normalized activity is less than or equal to that of the strain)/50. The panel maximum therefore corresponds to the 100th percentile (e.g., pectinase of CCNH008, 50/50), one strain ranking above gives the 98th percentile (49/50; e.g., α- and β-amylase and polyphenol oxidase), and mid-panel values give intermediate percentiles (e.g., cellulase, 29/50 = 58th). For enzymes not detected in CCNH008 (activity below the detection limit, recorded as zero), the tie-inclusive formula yields the percentile of the zero-value tie, and the enzyme is flagged as not detected (asterisks in Figure 4). Figure 4 displays the 19 percentiles as a ladder plot with bands: red, above the 75th percentile (13/19); gray, within the 25th–75th percentile or tied at zero because activity was not detected (5/19); green, below the 25th percentile (1/19). All percentile values are reproducible directly from Supplementary Table S2 using this rule.

4. Assay protocols, operational units, and calibration (Table S3-1)

Table S3-1 lists, for each of the 19 enzymes, the assay conditions, substrate and reaction, detection, the operational unit definition per gram fungal DW, and the protocol source; the calibration standard of every assay is assigned to its standard curve in Section 5 (Table S3-2).

Assay	Enzyme	Conditions	Substrate and reaction	Detection	Operational unit (per g DW)	Source

1	Pectinase	50 °C, pH 3.5	Pectin hydrolysed to galacturonic acid (reducing aldehyde groups react with DNS)	A540	1 mg galacturonic acid produced per hour	Patent (CN 121891417 A); DNS method [2]
2	Cellulase	37 °C, pH 5.5	Carboxymethyl cellulose sodium hydrolysed to reducing sugars; anthrone colorimetry	A620	1 µg reducing sugar per min	Patent (CN 121891418 A); anthrone [5]; cellulase framework [4]
3	Neutral protease	30 °C, pH 7.0	Casein hydrolysed to tyrosine; tyrosine reduces phosphomolybdic acid to tungsten blue	A680	1 nmol tyrosine-equivalent per min	Patent (CN 121891419 A); casein–Folin framework [3]
4	Alkaline protease	30 °C, pH 10.5	As assay 3	A680	1 nmol tyrosine-equivalent per min	Patent (CN 121891419 A); [3]
5	Acid protease	30 °C, pH 3.0	As assay 3	A680	1 nmol tyrosine-equivalent per min	Patent (CN 121891419 A); [3]
6	α-Amylase	70 °C, 15min	Starch α-1,4-glycosidic bonds → reducing sugars; DNS reaction	A540	1 mg reducing sugar per min; α-amylase is acid-labile	Patent (CN 121891417 A); DNS method [2]
7	β-Amylase	40 °C, pH 5.2	As assay 6	A540	1 mg reducing sugar per min	Patent (CN 121891417 A); [2]
8	Glucoamylase	40 °C, pH 4.6	Starch hydrolysed to glucose; DNS reaction	A540	1 mg glucose per min (in-house protocol)	This study
9	β-Glucosidase	37 °C, pH 5.0	p-Nitrophenyl-β-D-glucopyranoside → p-nitrophenol	A400	1 nmol p-nitrophenol per min	Patent (CN 121891417 A; CN 121891418 A)
10	α-Glucosidase	37 °C, pH 5.0	p-Nitrophenyl-α-D-glucopyranoside → p-nitrophenol	A400	1 nmol p-nitrophenol per min	Patent (CN 121891417 A; CN 121891418 A)
11	Acid xylanase	50 °C, pH 4.8	Xylan → reducing sugars; DNS reaction	A540	1 nmol reducing sugar per min	Patent (CN 120905040 B); [2]
12	Neutral xylanase	50 °C, pH 6.0	As assay 11	A540	1 nmol reducing sugar per min	Patent (CN 120905040 B); [2]
13	Alkaline xylanase	50 °C, pH 9.0	As assay 11	A540	1 nmol reducing sugar per min	Patent (CN 120905040 B); [2]
14	Polyphenol oxidase	25 °C, pH 6.0	Catechol oxidised to o-quinone	A525	ΔA525 = 0.01 per min per g per mL of reaction volume = 1 unit	Patent (CN 121891418 A); This study
15	Tannase	40 °C, pH 5.0	Propyl gallate (substrate) depletion; antioxidant substrate monitored before/after reaction	A270	0.01 µmol propyl gallate consumed per min	Patent (CN 121891418 A); This study
16	Feruloyl esterase	40 °C, pH 6.0	p-Nitrophenyl ferulate → p-nitrophenol + ferulate	A405	1 nmol p-nitrophenol per min	Patent (CN 121891418 A); pNP-ferulate method [6]
17	Glutaminase	37 °C, pH 7.0	Glutamine → L-glutamate + ammonia; Nessler's reagent	A420	1 nmol ammonia per min	Patent (CN 121891418 A); This study
18	Aminopeptidase	ELISA (double-antibody sandwich)	Purified anti-aminopeptidase capture antibody; crude enzyme; HRP-conjugated detection antibody; TMB substrate (yellow product)	A450	Signal proportional to aminopeptidase level in the sample (kit calibration)	Patent (CN 121891418 A); This study
19	Lipase	37 °C, pH 7.5	Olive oil hydrolysed to free fatty acids; copper-soap colorimetry	copper complex λmax	1 µmol fatty acid per min	Patent (CN 121891417 A); copper-soap method [7]

5. Calibration curves (Table S3-2)

#	Enzyme assay	X-axis	Y-axis	Linear equation	R^2
1	Amylase (α/β)	Standard concentration (mg/mL)	Absorbance	y = 2.481x − 0.1778	0.9965
2	Glucosidase (α/β)	Standard concentration (nmol/mL)	Absorbance	y = 0.0039x + 0.0027	0.998
3	Tannase	PG content (μmol/L)	Absorbance	y = 0.0029x + 0.0044	0.9994
4	Feruloyl esterase	PNP (nmol)	Absorbance	y = 0.0039x − 0.0132	0.9941
5	Glutaminase	Standard concentration (μmol/mL)	Absorbance	y = 1.524x − 0.0057	0.9983
6	Acid xylanase	Standard concentration (mg/mL)	Absorbance	y = 1.2777x − 0.0002	0.9983
7	Pectinase	Standard concentration (mg/mL)	Absorbance	y = 1.9821x − 0.008	0.9991
8	Cellulase	Standard concentration (mg/mL)	Absorbance	y = 2.509x − 0.0462	0.9993
9	Glucoamylase	Standard concentration (mg/mL)	Absorbance	y = 0.2164x − 0.0182	0.9989
10	Aminopeptidase	Standard concentration (U/mL)	Absorbance	y = 0.4635x − 0.4367	0.9829
11	Neutral xylanase	Standard concentration (mg/mL)	Absorbance	y = 0.8452x + 0.0058	0.9999
12	Alkaline xylanase	Standard concentration (mg/mL)	Absorbance	y = 1.4216x − 0.0293	0.9983
13	Protease (neutral / alkaline / acid)	L-tyrosine concentration (μg/mL)	Absorbance	y = 2.0021x − 0.0791	0.9989
14	Lipase	Oleic acid amount (μmol)	Absorbance	y = 1.9216x − 0.1783	0.9971

The three protease assays (Table S3-1, assays 3–5) share one L-tyrosine standard curve: tyrosine released from casein reduces phosphomolybdic acid to tungsten blue (A680), and the calibration relates A680 to the tyrosine concentration, as specified in CN 121891419 A and following the standard casein–Folin framework [3, 8]. The lipase assay (assay 19) is calibrated with an oleic-acid standard curve, measured as the copper–fatty-acid complex at 710 nm [7]. Polyphenol oxidase (assay 14) is a kinetic rate assay whose unit is defined directly by the rate ΔA525 = 0.01 per min per g per mL of reaction volume and therefore uses no external calibration standard.

6. References

[2] Miller GL. Use of dinitrosalicylic acid reagent for determination of reducing sugar. Anal Chem. (1959) 31(3):426–428. doi: 10.1021/ac60147a030.

[3] Anson ML. The estimation of pepsin, trypsin, papain, and cathepsin with hemoglobin. J Gen Physiol. (1938) 22(1):79–89. doi: 10.1085/jgp.22.1.79.

[4] Wood TM, Bhat KM. Methods for measuring cellulase activities. Methods Enzymol. (1988) 160:87–112. doi: 10.1016/0076-6879(88)60109-1.

[5] Dreywood R. Qualitative test for carbohydrate material. Ind Eng Chem Anal Ed. (1946) 18(8):499.

[6] Mastihuba V, Kremnický L, Mastihubová M, Willett JL, Côté GL. A spectrophotometric assay for feruloyl esterases. Anal Biochem. (2002) 309:96–101. doi: 10.1016/S0003-2697(02)00241-5.

[7] Kwon DY, Rhee JS. A simple and rapid colorimetric method for determination of free fatty acids for lipase assay. J Am Oil Chem Soc. (1986) 63(1):89–92. doi: 10.1007/BF02676129.

[8] Cupp-Enyard C. Sigma's non-specific protease activity assay - casein as a substrate. J Vis Exp. (2008) 19:e899. doi: 10.3791/899
