# Supplementary material for: Safety Profile and Enzymatic Specificity of Aspergillus cristatus CCNH008 Characterized by Genomic Analysis
Source: J Fungi (Basel). 2026 Oct 9;12(10):759. doi: 10.3390/jof12100759 (PMC13706197; doi:10.3390/jof12100759)
Supplement: Supplementary file 1 [file jof-12-00759-s001.zip › Table S6.ortholog_analysis.pdf]

Supplementary Table S6. Ortholog clustering, family expansion/contraction and pairwise dN/dS of pectinase-related genes in *Aspergillus cristatus* CCNH008 and four reference Aspergillus species.

All protein sets were clustered from an all-vs-all search (DIAMOND v2.2.7, E ≤ 0.001) followed by Markov-cluster (MCL) grouping; CAZy-family labels were assigned uniformly with hmmscan against the dbCAN HMM database V9; pairwise dN/dS was estimated with the Nei–Gojobori method (Jukes–Cantor correction); codon pairs with gaps or more than one nucleotide difference were excluded.

S6-1 Ortholog clustering and uniform CAZyme annotation summary

Species	Proteins	Genes in orthogroups	Orthogroups containing species	CAZyme domain rows (dbCAN V9)	CAZyme genes
A. cristatus CCNH008	10599	10592	8788	1703	592
A. niger CBS 513.88	13872	13863	11966	3042	936
A. oryzae RIB40	12074	12074	9870	3623	1087
A. nidulans FGSC A4	9561	9560	8163	3059	910
A. fumigatus Af293	9630	9629	8458	3148	831

Total orthogroups: 22,784; 8,516 orthogroups contain more than one gene, and 8,139 contain genes from more than one species.

S6-2 CAZy-family expansion/contraction in CCNH008 (Fisher exact test vs the gene background of the other four species, BH-FDR corrected)

Family	CCNH008 genes	Other 4 species (pooled)	Others (mean/species)	Direction	Odds ratio	P	q (BH)	Significant
AA1	13	41	10.2	expanded	2.039	0.042	1.0	no
AA9	8	44	11.0	contracted	1.158	0.683	1.0	no
CE1	36	321	80.2	contracted	0.694	0.0438	1.0	no
CE12	1	19	4.8	contracted	0.334	0.507	1.0	no
CE8	0	15	3.8	contracted	0.0	0.247	1.0	no
GH105	6	30	7.5	contracted	1.274	0.622	1.0	no
GH12	2	13	3.2	contracted	0.978	1	1.0	no
GH13	17	98	24.5	contracted	1.106	0.679	1.0	no
GH28	3	67	16.8	contracted	0.281	0.0206	1.0	no
GH35	1	33	8.2	contracted	0.191	0.0772	1.0	no
GH43	12	93	23.2	contracted	0.817	0.665	1.0	no
GH53	1	5	1.2	contracted	1.272	0.584	1.0	no
GH6	0	6	1.5	contracted	0.0	1	1.0	no
GH7	1	14	3.5	contracted	0.453	0.709	1.0	no
GH78	6	44	11.0	contracted	0.866	1	1.0	no
PL1	4	47	11.8	contracted	0.538	0.304	1.0	no
PL3	4	23	5.8	contracted	1.106	0.779	1.0	no
PL4	2	19	4.8	contracted	0.668	1	1.0	no

Families shown: all pectinase-relevant families plus every significantly ($q < 0.05$) expanded/contracted family (0 of 327 families significant in total). Direction refers to the gene count of CCNH008 relative to the per-species mean of the other four species.

S6-3 Pairwise dN/dS (Nei–Gojobori, Jukes–Cantor corrected) of CCNH008 pectinase-related genes and their best-matching orthologs

Family	Reference species	CCNH008 gene	Ortholog	Protein identity (%)	dN	dS	dN/dS
CE1	A. oryzae	g236	XP_001824982.1	43.2	0.0164	0.1801	0.0908
GH78	A. oryzae	g419	XP_001816708.1	63.3	0.0075	0.2442	0.0306
CE1	A. oryzae	g465	XP_023093605.1	39.5	0.0108	0.124	0.0873
CE1	A. oryzae	g673	XP_001821143.3	38.0	0.0106	0.1653	0.064
CE1	A. oryzae	g737	XP_001817948.1	48.6	0.0196	0.1623	0.1211
GH105	A. oryzae	g761	XP_001820300.1	40.3	0.0134	0.2206	0.0609
GH53	A. oryzae	g1126	XP_001819006.1	63.5	0.0147	0.1851	0.0792
CE1	A. oryzae	g1411	XP_001727401.3	37.7	0.0157	0.1503	0.1047
CE1	A. oryzae	g1569	XP_023090162.1	70.6	0.0102	0.1974	0.0515
CE1	A. oryzae	g1842	XP_001817852.1	71.2	0.0109	0.2205	0.0494
GH78	A. oryzae	g1940	XP_001820356.1	69.6	0.0136	0.2089	0.065
PL1	A. oryzae	g1945	XP_023090297.1	55.1	0.012	0.162	0.0743
PL1	A. oryzae	g1989	XP_001823182.1	60.6	0.0087	0.1458	0.0597
GH43	A. oryzae	g2015	XP_001823245.1	58.9	0.0137	0.1911	0.0715
CE1	A. oryzae	g2092	XP_001817268.1	45.3	0.0144	0.2678	0.0537
GH43	A. oryzae	g2403	XP_023089311.1	53.4	0.0143	0.2082	0.0689
GH43	A. oryzae	g2686	XP_023089311.1	46.3	0.0156	0.203	0.0767
PL1	A. oryzae	g3097	XP_001827132.1	49.4	0.0128	0.2004	0.0639
CE1	A. oryzae	g3474	XP_023088695.1	63.6	0.011	0.2098	0.0523
CE1	A. oryzae	g3479	XP_023090936.1	45.9	0.0163	0.1825	0.089

Family	Reference species	CCNH008 gene	Ortholog	Protein identity (%)	dN	dS	dN/dS
GH105	A. oryzae	g3691	XP_001822282.1	50.0	0.0178	0.1497	0.1187
GH35	A. oryzae	g4237	XP_001727461.1	72.9	0.0117	0.2218	0.0528
CE1	A. oryzae	g4366	XP_023089259.1	46.7	0.0107	0.2404	0.0447
PL1	A. oryzae	g4656	XP_001820953.1	67.7	0.0197	0.1121	0.1754
GH105	A. oryzae	g5034	XP_023092626.1	46.1	0.0144	0.154	0.0932
CE1	A. oryzae	g5101	XP_001825690.1	67.6	0.013	0.212	0.0614
CE1	A. oryzae	g5184	XP_001817733.1	56.4	0.0101	0.2326	0.0436
CE1	A. oryzae	g5236	XP_001826653.1	61.3	0.0126	0.2	0.0629
CE1	A. oryzae	g5350	XP_001822738.1	74.8	0.0112	0.2377	0.047
CE1	A. oryzae	g5444	XP_001817361.1	61.0	0.0084	0.2438	0.0345
PL3	A. oryzae	g5570	XP_001817532.1	27.2	0.0173	0.1457	0.1191
PL3	A. oryzae	g5574	XP_001817532.1	29.8	0.0225	0.1338	0.1678
CE1	A. oryzae	g5812	XP_001818403.3	38.8	0.0154	0.1974	0.0779
CE1	A. oryzae	g5900	XP_001824552.3	49.8	0.0153	0.182	0.0842
GH28	A. oryzae	g6161	XP_001825455.1	21.7	0.0119	0.2026	0.0587
GH28	A. oryzae	g6162	XP_001825455.1	47.2	0.0137	0.1889	0.0727
CE1	A. oryzae	g7297	XP_023088914.1	31.0	0.0083	0.1956	0.0424
CE1	A. oryzae	g7337	XP_001822064.1	68.0	0.0139	0.1434	0.0973
GH78	A. oryzae	g7410	XP_023089509.1	64.8	0.0124	0.2198	0.0564
CE1	A. oryzae	g7450	XP_023093605.1	46.0	0.007	0.1308	0.0538
CE1	A. oryzae	g7849	XP_001823105.1	59.6	0.0068	0.2726	0.0248
CE12	A. oryzae	g8199	XP_001823386.1	36.9	0.0176	0.1413	0.1245
GH43	A. oryzae	g8208	XP_023092157.1	16.0	0.0117	0.195	0.0602
GH105	A. oryzae	g8244	XP_001818725.1	79.6	0.0079	0.2381	0.0332
CE1	A. oryzae	g8248	XP_023090488.1	49.8	0.015	0.1597	0.0936
CE1	A. oryzae	g8260	XP_001727401.3	39.6	0.0201	0.1521	0.1323
CE1	A. oryzae	g8348	XP_001819091.1	61.1	0.0089	0.2027	0.0437
CE1	A. oryzae	g8480	XP_001819363.3	24.4	0.0134	0.157	0.0853
PL4	A. oryzae	g9067	XP_001825997.1	32.3	0.0099	0.2432	0.0406
GH43	A. oryzae	g9199	XP_001822207.1	71.1	0.0107	0.2347	0.0458
GH78	A. oryzae	g9408	XP_023092009.1	43.5	0.0123	0.1882	0.0654
GH105	A. oryzae	g9750	XP_001826713.1	59.2	0.0119	0.2166	0.0551
GH43	A. oryzae	g9771	XP_023090474.1	43.1	0.0145	0.1676	0.0866
GH43	A. oryzae	g9823	XP_001823953.1	40.5	0.0093	0.2146	0.0432
CE1	A. oryzae	g10116	XP_023092442.1	44.2	0.011	0.2018	0.0543
GH105	A. oryzae	g10132	XP_001820464.1	50.6	0.0142	0.176	0.0805
CE1	A. oryzae	g10247	XP_001817733.1	31.5	0.0203	0.1139	0.1779
CE1	A. oryzae	g10271	XP_001817948.1	51.1	0.0139	0.1983	0.0699
GH43	A. oryzae	g10375	XP_001823666.1	75.3	0.0135	0.1735	0.0778
GH78	A. nidulans	g419	EAA62728.1	60.6	0.0098	0.2454	0.04
CE1	A. nidulans	g465	EAA63018.1	48.9	0.0144	0.1741	0.0827
CE1	A. nidulans	g673	EAA59511.1	28.8	0.0145	0.1199	0.1207
GH105	A. nidulans	g761	EAA64877.1	40.3	0.0129	0.2138	0.0603
GH53	A. nidulans	g1126	EAA62820.1	70.8	0.0108	0.1987	0.0544
CE1	A. nidulans	g1569	EAA59517.1	67.8	0.0087	0.235	0.0368
CE1	A. nidulans	g1842	EAA65719.1	69.2	0.0122	0.2074	0.0589
PL1	A. nidulans	g1945	EAA60960.1	53.7	0.0142	0.1366	0.1043
PL1	A. nidulans	g1989	EAA61832.1	53.8	0.0121	0.1528	0.079
CE1	A. nidulans	g2092	EAA59965.1	46.5	0.0127	0.2414	0.0525
GH43	A. nidulans	g2403	EAA63615.1	43.7	0.0192	0.163	0.118
GH43	A. nidulans	g2686	EAA63615.1	41.6	0.0172	0.1748	0.0983
CE1	A. nidulans	g2776	EAA66397.1	36.2	0.0171	0.1087	0.1569
PL1	A. nidulans	g3097	EAA64442.1	53.3	0.012	0.1432	0.0837
CE1	A. nidulans	g3479	EAA61774.1	62.6	0.0124	0.2062	0.0602
GH105	A. nidulans	g3691	EAA58494.1	46.0	0.0189	0.1562	0.1211
GH35	A. nidulans	g4237	EAA65398.1	65.9	0.0105	0.2119	0.0496
CE1	A. nidulans	g4366	EAA65904.1	43.6	0.0107	0.2475	0.0434
PL1	A. nidulans	g4656	EAA60289.1	56.5	0.0188	0.1215	0.1551
GH105	A. nidulans	g5034	EAA66450.1	31.1	0.0187	0.1142	0.164
CE1	A. nidulans	g5101	EAA63211.1	62.0	0.0125	0.1709	0.0731
CE1	A. nidulans	g5184	EAA64677.1	30.9	0.0161	0.1099	0.1469
CE1	A. nidulans	g5236	EAA64563.1	53.9	0.0145	0.1801	0.0805
CE1	A. nidulans	g5292	EAA58824.1	24.1	0.0173	0.141	0.1228
CE1	A. nidulans	g5350	EAA58980.1	73.0	0.0121	0.2202	0.055
CE1	A. nidulans	g5444	EAA60575.1	56.9	0.0105	0.1896	0.0556
PL3	A. nidulans	g5570	EAA67075.1	27.9	0.0128	0.1348	0.0947
PL3	A. nidulans	g5574	EAA64647.1	35.4	0.0142	0.1432	0.0994
CE1	A. nidulans	g5812	EAA63517.1	40.6	0.0138	0.21	0.0655
CE1	A. nidulans	g5900	EAA60068.1	62.7	0.0142	0.1787	0.0795
GH28	A. nidulans	g6161	EAA61967.1	20.8	0.0115	0.1964	0.0585
GH28	A. nidulans	g6162	EAA61967.1	42.2	0.0087	0.1931	0.0451
GH78	A. nidulans	g6969	EAA67054.1	50.4	0.0114	0.1975	0.0578
CE1	A. nidulans	g7297	EAA61462.1	27.4	0.0139	0.145	0.0962

Family	Reference species	CCNH008 gene	Ortholog	Protein identity (%)	dN	dS	dN/dS
CE1	A. nidulans	g7337	EAA60874.1	33.5	0.0129	0.1881	0.0688
GH78	A. nidulans	g7410	EAA62978.1	36.7	0.0188	0.1721	0.1092
CE1	A. nidulans	g7450	EAA63018.1	56.6	0.0178	0.2233	0.0796
CE1	A. nidulans	g7849	EAA63914.1	58.4	0.008	0.2679	0.0298
CE12	A. nidulans	g8199	EAA64634.1	36.5	0.0176	0.1716	0.1025
GH105	A. nidulans	g8244	EAA62960.1	71.7	0.008	0.2155	0.0369
CE1	A. nidulans	g8248	EAA58411.1	50.2	0.0125	0.1609	0.078
CE1	A. nidulans	g8348	EAA63948.1	56.1	0.0143	0.176	0.0815
CE1	A. nidulans	g8480	EAA59282.1	36.0	0.0145	0.1422	0.1021
PL4	A. nidulans	g9067	EAA61387.1	32.1	0.0091	0.228	0.04
GH78	A. nidulans	g9408	EAA58135.1	37.4	0.0151	0.1936	0.0778
GH105	A. nidulans	g9750	EAA67043.1	52.7	0.016	0.175	0.0914
GH43	A. nidulans	g9771	EAA58736.1	34.8	0.015	0.1301	0.1151
GH43	A. nidulans	g9823	EAA66990.1	39.1	0.0085	0.2232	0.038
CE1	A. nidulans	g10116	EAA60541.1	41.0	0.0176	0.1626	0.1084
GH105	A. nidulans	g10132	EAA63396.1	30.8	0.0176	0.169	0.1041
CE1	A. nidulans	g10247	EAA64677.1	51.3	0.0147	0.1647	0.0894
GH43	A. nidulans	g10375	EAA62979.1	76.2	0.0089	0.1895	0.047
GH78	A. fumigatus	g419	EAL89440.1	60.5	0.0106	0.2156	0.0491
CE1	A. fumigatus	g465	EAL86808.1	40.2	0.0158	0.1123	0.1404
CE1	A. fumigatus	g673	EAL86207.1	28.3	0.0183	0.1203	0.1518
CE1	A. fumigatus	g737	EAL84656.1	27.2	0.0151	0.1203	0.1258
GH105	A. fumigatus	g761	EAL89781.1	36.9	0.0137	0.2161	0.0635
GH53	A. fumigatus	g1126	EAL88402.1	64.3	0.0104	0.2002	0.0517
CE1	A. fumigatus	g1569	EAL88101.1	73.2	0.0065	0.2406	0.0272
CE1	A. fumigatus	g1842	EAL87970.1	69.4	0.0122	0.2167	0.0563
GH78	A. fumigatus	g1940	EAL87878.1	63.6	0.017	0.1675	0.1014
PL1	A. fumigatus	g1945	EAL91566.1	35.7	0.0182	0.0926	0.1967
PL1	A. fumigatus	g1989	EAL87175.1	55.0	0.0131	0.1394	0.094
GH43	A. fumigatus	g2015	EAL87222.1	56.6	0.0165	0.152	0.1085
CE1	A. fumigatus	g2092	EAL87006.1	46.3	0.0076	0.264	0.0287
GH43	A. fumigatus	g2403	EAL91062.1	44.7	0.0153	0.1524	0.1006
GH43	A. fumigatus	g2686	EAL91062.1	39.0	0.019	0.142	0.1339
CE1	A. fumigatus	g2776	EAL93599.1	65.3	0.0164	0.1875	0.0872
PL1	A. fumigatus	g3097	EAL91566.1	50.1	0.0143	0.1683	0.0852
CE1	A. fumigatus	g3479	EAL84987.2	51.9	0.0129	0.1904	0.0676
GH105	A. fumigatus	g3691	EAL87167.1	47.0	0.0194	0.1395	0.1393
GH35	A. fumigatus	g4237	EAL90749.1	69.1	0.0123	0.2	0.0616
CE1	A. fumigatus	g4366	EAL90865.1	42.4	0.0154	0.2368	0.065
PL1	A. fumigatus	g4656	EAL89402.1	75.0	0.0132	0.1373	0.0958
GH105	A. fumigatus	g5034	EAL93757.1	32.9	0.0165	0.1109	0.1484
CE1	A. fumigatus	g5184	EAL85241.2	58.8	0.0096	0.2294	0.042
CE1	A. fumigatus	g5236	EAL85190.1	58.0	0.0147	0.1935	0.0762
CE1	A. fumigatus	g5350	EAL85929.1	75.6	0.0126	0.2183	0.0576
CE1	A. fumigatus	g5444	EAL91616.1	61.3	0.0077	0.2028	0.0381
PL3	A. fumigatus	g5570	EAL86832.1	28.2	0.0139	0.1303	0.1065
PL3	A. fumigatus	g5574	EAL87824.1	30.1	0.0189	0.1407	0.134
CE1	A. fumigatus	g5812	EAL92790.2	40.8	0.0146	0.1922	0.076
CE1	A. fumigatus	g5900	EAL92844.1	69.2	0.0152	0.2139	0.071
GH28	A. fumigatus	g6161	EAL91553.1	19.0	0.0182	0.1739	0.1045
GH28	A. fumigatus	g6162	EAL91553.1	41.0	0.0049	0.2043	0.0238
CE1	A. fumigatus	g6709	EAL88042.1	41.0	0.0204	0.1487	0.1375
CE1	A. fumigatus	g7297	EAL94052.1	31.4	0.0157	0.1498	0.1049
CE1	A. fumigatus	g7337	EAL87389.1	64.7	0.0156	0.1629	0.096
GH78	A. fumigatus	g7410	EAL86572.1	67.3	0.0102	0.2075	0.049
CE1	A. fumigatus	g7450	EAL86808.1	43.2	0.0112	0.1574	0.0709
CE1	A. fumigatus	g7849	EAL91875.1	60.7	0.0068	0.2617	0.026
CE12	A. fumigatus	g8199	EAL92136.1	39.8	0.0133	0.1676	0.0793
GH43	A. fumigatus	g8208	EAL92128.1	36.1	0.0095	0.177	0.0536
GH105	A. fumigatus	g8244	EAL84434.1	79.6	0.0071	0.237	0.0299
CE1	A. fumigatus	g8248	EAL88930.1	51.2	0.0154	0.1629	0.0944
CE1	A. fumigatus	g8348	EAL88754.1	59.6	0.0097	0.1948	0.05
CE1	A. fumigatus	g8480	EAL88650.1	24.6	0.0154	0.174	0.0883
PL4	A. fumigatus	g9067	EAL84527.1	30.9	0.0111	0.1808	0.0614
GH43	A. fumigatus	g9199	EAL93769.1	72.0	0.0084	0.2134	0.0394
GH78	A. fumigatus	g9408	EAL85619.1	41.3	0.0123	0.2084	0.0588
GH105	A. fumigatus	g9750	EAL86486.1	52.2	0.0139	0.1778	0.0781
GH43	A. fumigatus	g9771	EAL93710.1	35.7	0.0204	0.1362	0.1497
GH43	A. fumigatus	g9823	EAL91739.1	40.6	0.0104	0.2109	0.0495
CE1	A. fumigatus	g10116	EAL85761.1	41.9	0.0176	0.2068	0.0852
GH105	A. fumigatus	g10132	EAL84356.1	52.6	0.0127	0.1827	0.0696
CE1	A. fumigatus	g10247	EAL93199.1	56.7	0.0122	0.1718	0.0708
CE1	A. fumigatus	g10271	EAL84656.1	34.6	0.0143	0.1484	0.0967

n = 166 orthologous pairs; median dN/dS = 0.074; maximum = 0.197; no pair exceeded 1, i.e. all retained pectinase-related genes are under purifying selection and no coding-level positive selection was detected. Tandem-duplication screening found no tandem arrays (≥ 2 adjacent same-family genes) for GH28 (3 genes), PL1 (4), PL3 (4), PL4 (2), GH43 (12) or CE1 (36); the expanded debranching genes are scattered singly across the genome.
